# Structural basis for the recognition of muramyltripeptide by *Helicobacter pylori* Csd4, a d,l-carboxypeptidase controlling the helical cell shape

**DOI:** 10.1107/S1399004714018732

**Published:** 2014-10-16

**Authors:** Hyoun Sook Kim, Jieun Kim, Ha Na Im, Doo Ri An, Mijoon Lee, Dusan Hesek, Shahriar Mobashery, Jin Young Kim, Kun Cho, Hye Jin Yoon, Byung Woo Han, Byung Il Lee, Se Won Suh

**Affiliations:** a Research Institute of Pharmaceutical Sciences, College of Pharmacy, Seoul National University, Seoul 151-742, Republic of Korea; b Department of Chemistry, College of Natural Sciences, Seoul National University, Seoul 151-742, Republic of Korea; c Department of Biophysics and Chemical Biology, College of Natural Sciences, Seoul National University, Seoul 151-742, Republic of Korea; d Department of Chemistry and Biochemistry, University of Notre Dame, Notre Dame, IN 46556, USA; e Division of Mass Spectrometry, Korea Basic Science Institute, Chungbuk 363-883, Republic of Korea; f Biomolecular Function Research Branch, Division of Convergence Technology, Research Institute, National Cancer Center, Gyeonggi 410-769, Republic of Korea

**Keywords:** *csd4*, *csd5*, HP1075, d,l-carboxypeptidase, *Helicobacter pylori*, peptidoglycan, *meso*-diaminopimelate, *pgp1*, cell shape

## Abstract

*Helicobacter pylori* infection causes a variety of gastrointestinal diseases, including peptic ulcers and gastric cancer. Its colonization of the gastric mucosa of the human stomach is a prerequisite for survival in the stomach. Colonization depends on its motility, which is facilitated by the helical shape of the bacterium. In *H. pylori*, cross-linking relaxation or trimming of peptidoglycan muropeptides affects the helical cell shape. Csd4 has been identified as one of the cell shape-determining peptidoglycan hydrolases in *H. pylori*. It is a Zn^2+^-dependent d,l-carboxypeptidase that cleaves the bond between the γ-d-Glu and the *m*DAP of the non-cross-linked muramyl­tripeptide (muramyl-l-Ala-γ-d-Glu-*m*DAP) of the peptidoglycan to produce the muramyldipeptide (muramyl-l-Ala-γ-d-Glu) and *m*DAP. Here, the crystal structure of *H. pylori* Csd4 (HP1075 in strain 26695) is reported in three different states: the ligand-unbound form, the substrate-bound form and the product-bound form. *H. pylori* Csd4 consists of three domains: an N-terminal d,l-carboxypeptidase domain with a typical carboxy­peptidase fold, a central β-barrel domain with a novel fold and a C-terminal immunoglobulin-like domain. The d,l-carboxypeptidase domain recognizes the substrate by interacting primarily with the terminal *m*DAP moiety of the muramyltripeptide. It undergoes a significant structural change upon binding either *m*DAP or the *m*DAP-containing muramyl­tripeptide. It it also shown that Csd5, another cell-shape determinant in *H. pylori*, is capable of interacting not only with *H. pylori* Csd4 but also with the dipeptide product of the reaction catalyzed by Csd4.

## Abstract 2

*H. pylori* Csd4 (HP1075), together with other peptidoglycan hydrolases, plays an important role in determining the helical cell shape. Its crystal structure has been determined in three different forms.

## 1. Introduction   

*Helicobacter pylori* infects approximately half of the world’s population. Infection by *H. pylori* can cause a variety of upper gastrointestinal disorders such as chronic gastritis, gastric and duodenal ulcers, gastric adenocarcinoma and gastric mucosa-associated lymphoid tissue (MALT) lymphoma (Kusters *et al.*, 2006 ▶). A triple therapy consisting of a proton-pump inhibitor such as omeprazole and the antibiotics clarithromycin and amoxicillin (or metronidazole) is effective in the eradication of *H. pylori* in most cases. However, increasing antibiotic resistance requires new therapies and the discovery of new antibiotics (Malfertheiner *et al.*, 2012 ▶).

High motility of *H. pylori* is required for its colonization of the human stomach and allows the bacterium to survive in its preferred niche, the gastric mucosa (Ottemann & Lowenthal, 2002 ▶). The helical cell shape of *H. pylori* is thought to facilitate efficient colonization of the viscous epithelial mucus layer *via* a corkscrewing mechanism (Berg & Turner, 1979 ▶). Several cell-shape mutants that have lost the helical twist and/or curvature exhibit attenuated colonization (Wyckoff *et al.*, 2012 ▶). The peptidoglycan layer of the bacterial cell wall not only withstands the turgor pressure as a stress-bearing structure but also maintains the cell shape (Vollmer & Bertsche, 2008 ▶; Sycuro *et al.*, 2010 ▶). The essential component of peptidoglycan consists of a linear polysaccharide chain, which is a polymer of alternating *N*-acetylglucosamine (NAG) and *N*-acetylmuramic acid (NAM) disaccharides, with a pentapeptide linked to NAM (Vollmer, Blanot *et al.*, 2008 ▶). In *H. pylori* the pentapeptide sequence is l-Ala^1^-γ-d-Glu^2^-*m*DAP^3^-d-Ala^4^-d-Ala^5^, where *m*DAP refers to *meso*-2,6-diaminopimelate. The *Helicobacter* peptidoglycan layer is cross-linked exclusively by 4→3 linkages (cross-links from the side chain of *m*DAP^3^ from one strand to the main-chain d-Ala^4^ of another; Costa *et al.*, 1999 ▶) to form a mesh-like structure termed peptidoglycan sacculus (Meroueh *et al.*, 2006 ▶).

In many bacteria, the peptidoglycan layer is remodelled by a number of cell-wall hydrolases as well as synthetases for peptidoglycan maturation, regulation of cell-wall growth, cell division, peptidoglycan turnover and recycling, cell lysis and the release of peptidoglycan fragments for host–pathogen interactions (Vollmer, Joris *et al.*, 2008 ▶). In *H. pylori*, seven cell shape-determining genes have been identified to date: *csd1*, *csd2*, *csd3*/*hdpA*, *ccmA*, *csd4*,* csd5* and *csd6* (Sycuro *et al.*, 2010 ▶, 2012 ▶, 2013 ▶; Bonis *et al.*, 2010 ▶; Frirdich *et al.*, 2012 ▶). Peptidoglycan cross-linking relaxation by these proteins is critical for the helical cell shape of *H. pylori* and for stomach colonization. *H. pylori* Csd1, Csd2 and Csd3/HdpA proteins belong to the M23B/LytM family of metallopeptidases and show d,d-endopeptidase (d,d-EPase) activity (Sycuro *et al.*, 2010 ▶; Bonis *et al.*, 2010 ▶). Csd3/HdpA has an additional d,d-carboxypeptidase (d,d-CPase) activity (Bonis *et al.*, 2010 ▶). CcmA does not encode a putative enzyme and CcmA-like bactofilins form cytoplasmic filaments that bind a peptido­glycan-synthesis enzyme for localized activity (Koch *et al.*, 2011 ▶; Kühn *et al.*, 2010 ▶).

Csd4 is a Zn^2+^-dependent d,l-carboxypeptidase (d,l-CPase) which cleaves the γ-d-Glu^2^-*m*DAP^3^ bond of the non-cross-linked peptidoglycan muramyltripeptide (muramyl-l-Ala^1^-γ-d-Glu^2^-*m*DAP^3^) to produce the corresponding muramyl­dipeptide (muramyl-l-Ala-γ-d-Glu) and *m*DAP. On the other hand, Csd5 is a putative scaffolding protein that appears to be unique to *H. pylori* and closely related bacterial species (Sycuro *et al.*, 2012 ▶). The deletion mutant of *csd4* (Δ*csd4*) loses nearly all curvature, yielding a straight rod, despite having a normal growth rate (Sycuro *et al.*, 2012 ▶). The curvature-inducing function of Csd4 does not depend on the M23B/LytM EPase activity of Csd1–Csd3 (Sycuro *et al.*, 2012 ▶). The Δ*csd*4 mutant with the straight rod cell shape shows impaired stomach colonization (Sycuro *et al.*, 2012 ▶). A Csd4 homologue in *Campylobacter jejuni*, Pgp1, exhibits a similar Zn^2+^-dependent d,l-CPase activity against the monomeric disaccharide tripeptide substrate and promotes the helical rod shape of this organism (Frirdich *et al.*, 2012 ▶). It has 41% sequence identity and 60% similarity to *H. pylori* Csd4. These studies suggest that the d,l-CPase activity of Csd4 (or of its close homologues) is vital for generating the proper cell shape and colonization of helically shaped bacteria. In addition, Csd5 was identified as another cell-shape determinant (Sycuro *et al.*, 2012 ▶) which does not contain any known enzymatic domain but does contain a bacterial SH3 domain that could allow interactions with other peptidoglycan peptidases or with peptidoglycan itself. It was suggested that Csd4 and Csd5 act at a similar stage of helical cell-shape specification (Sycuro *et al.*, 2012 ▶). The *csd4csd5* double mutant resembled the *csd4* mutant both in global peptidoglycan changes and in having a straighter shape than the *csd5* mutant (Sycuro *et al.*, 2012 ▶), suggesting that Csd5 acts downstream of Csd4.

Despite the importance of these cell shape-regulating proteins of *H. pylori* in promoting stomach colonization for its survival, no structural studies on them have been reported. To provide the structural basis for understanding the molecular function of *H. pylori* Csd4, we have determined its crystal structure in three different states: (i) a ligand-free form (Csd4-unbound), (ii) a substrate-bound form (Csd–muramyltripeptide) complexed with the synthetic peptidoglycan fragment NAM-tripeptide (NAM-l-Ala-γ-d-Glu-*m*DAP) and (iii) a product-bound form (Csd4–*m*DAP) complexed with *m*DAP, one of the two products. *H. pylori* Csd4 consists of three domains: an N-terminal CPase domain (residues 22–267), a central β-barrel domain with a novel fold (residues 268–340) and a C-terminal immunoglublin-like domain (residues 341–438). Our structures reveal that the CPase domain of *H. pylori* Csd4 primarily recognizes the terminal *m*DAP moiety of the muramyltripeptide substrate and undergoes a significant structural change upon binding either *m*DAP or the *m*DAP-containing tripeptide. We also show that *H. pylori* Csd5 directly interacts with *H. pylori* Csd4 and with the muramyldipeptide generated by the enzymatic activity of Csd4. The structures disclosed herein could serve as a foundation for the discovery of novel inhibitors that would prove helpful in fighting infections by drug-resistant *H. pylori*.

## 2. Materials and methods   

### 2.1. Protein expression and purification   

The N-terminal 19 residues of Csd4 (HP1075) from *H. pylori* strain 26695 are predicted to form a signal peptide by the *SignalP* 4.1 server (Petersen *et al.*, 2011 ▶). Therefore, we PCR-amplified the gene covering residues Met22–Val438 of Csd4 and cloned it into the pET-28a(+) vector (Novagen) using *Nde*I and *Xho*I restriction enzymes. The resulting recombinant Csd4 protein is fused with a His_6_-containing tag (MGSSHHHHHHSSGLVPRGSH) at its N-terminus. It was expressed in *Escherichia coli* Rosetta2(DE3) cells using Luria Broth culture medium. Protein expression was induced using 0.5 m*M* isopropyl β-d-1-thiogalactopyranoside and the cells were incubated for an additional 15 h at 30°C following growth to mid-log phase at 37°C. The cells were lysed by sonication in buffer *A* (50 m*M* Tris–HCl pH 7.9, 500 m*M* sodium chloride, 50 m*M* imidazole) containing 5%(*v*/*v*) glycerol and 1 m*M* phenylmethylsulfonyl fluoride. The crude lysate was centrifuged at 36 000*g* for 1 h. The supernatant was applied onto a HiTrap Chelating HP affinity-chromatography column (GE Healthcare) which had previously been equilibrated with buffer *A*. Upon eluting with an imidazole gradient in the same buffer, the Csd4 protein eluted at 250–300 m*M* imidazole concentration. The eluted protein was diluted fivefold with buffer *B* (20 m*M* sodium phosphate pH 6.0) and was applied onto a HiTrap SP HP column (GE Healthcare) which had previously been equilibrated with buffer *B* containing 100 m*M* sodium chloride. Upon eluting with a gradient of sodium chloride in buffer *B*, the protein was eluted at 450–500 m*M* sodium chloride concentration. The eluted protein was further purified using a HiLoad XK-16 Superdex 200 column (GE Healthcare) which had previously been equilibrated with 20 m*M* Tris–HCl pH 7.0, 200 m*M* sodium chloride. Selenomethionine (SeMet)-substituted protein was overexpressed and purified essentially as above except that M9 cell-culture medium was used.

The N-terminal 40-residue-truncated construct of Csd5 (HP1250) from *H. pylori* strain 26695 was also designed based on prediction of the transmembrane region (Pro15–Val37). The gene covering residues Ser41–Glu192 was amplified and cloned into the pET-28b(+) vector (Novagen) using *Nde*I and *Xho*I restriction enzymes, producing the recombinant Csd5 protein with a His_6_-containing tag (MGSSHHHHH­HSSGLVPRGSHM) at its N-terminus. The Csd5 protein was overexpressed and purified essentially as above, except that the buffer used for gel filtration was 10 m*M* HEPES pH 7.5, 100 m*M* sodium chloride.

### 2.2. Crystallization and structure determination   

Fractions containing the recombinant Csd4 were pooled and concentrated to 8.3 mg ml^−1^ for crystallization. All crystals of Csd4 (including SeMet-labelled Csd4) were grown by the sitting-drop vapour-diffusion method at 23°C by mixing 0.4 µl each of the protein solution and a reservoir solution consisting of 200 m*M* calcium chloride, 100 m*M* HEPES pH 7.5, 25%(*w*/*v*) polyethylene glycol 3350. We could only obtain crystals in the presence of calcium ions in the reservoir. Rod-shaped crystals grew to 0.2 × 0.1 × 0.1 mm in a week. Serendipitously, we obtained crystals of uncomplexed Csd4 (Csd4-unbound) and of Csd4 bound to *m*DAP (Csd4–*m*DAP) under the same conditions. It appeared that *m*DAP was present in *E. coli* at a sufficient concentration and bound to Csd4 in a minor fraction of the crystals even when we did not intentionally add *m*DAP during the purification and crystallization steps. For data collection of Csd4-unbound and Csd4–*m*DAP, crystals were dipped into reservoir solution containing 20%(*v*/*v*) glycerol for 1–2 min and were flash-cooled using a cold nitrogen-gas stream at 100 K. To obtain crystals of the Csd4–muramyltripeptide complex, crystals of native Csd4 were dipped for 90 s into 1.5 µl reservoir solution supplemented with 20%(*v*/*v*) glycerol and 50 m*M* NAM-tripeptide and were flash-cooled in a cold nitrogen-gas stream at 100 K for data collection. The NAM-tripeptide was synthesized as described previously (Lee *et al.*, 2009 ▶).

Single-wavelength anomalous diffraction (SAD) data were collected to 1.60 Å resolution from a crystal of SeMet-substituted Csd4 (Table 1 ▶). X-ray data for Csd4-unbound, the Csd4–muramyltripeptide complex and the Csd4–*m*DAP complex were collected to 1.60, 1.55 and 1.41 Å resolution, respectively (Table 1 ▶). Raw X-ray diffraction data were processed and scaled using the *HKL*-2000 program suite (Otwinowski & Minor, 1997 ▶). SAD phases were calculated with *AutoSol* from the *PHENIX* software package (Adams *et al.*, 2010 ▶) and were further improved by the automatic model-building program *RESOLVE* (Terwilliger, 2003 ▶) to build an initial model. The model of SeMet-substituted Csd4 was completed using iterative cycles of model building with *Coot* (Emsley *et al.*, 2010 ▶) and refinement with *REFMAC*5 from the *CCP*4 suite (Murshudov *et al.*, 2011 ▶; Winn *et al.*, 2011 ▶). The structures of Csd4-unbound, Csd4–*m*DAP and Csd4–muramyltripeptide were determined by molecular replacement with *MOLREP* (Vagin & Teplyakov, 2010 ▶) using the structure of the SeMet-substituted Csd4. Stereochemistry of the refined models was evaluated using *MolProbity* (Chen *et al.*, 2010 ▶). Data-collection and refinement statistics are given in Table 1 ▶.

### 2.3. Identification of metal-binding sites by anomalous scattering   

To test whether the metal-binding sites can be occupied by zinc ions, crystals of Csd4–*m*DAP were soaked for 80 s in 3 µl of a solution consisting of 200 m*M* sodium chloride, 100 m*M* HEPES pH 7.5, 25%(*w*/*v*) polyethylene glycol 3350, 2 m*M* EDTA, 20%(*v*/*v*) glycerol and then soaked for 80 s in 3 µl of a Zn^2+^-containing solution [100 m*M* zinc chloride, 100 m*M* sodium chloride, 100 m*M* HEPES pH 7.5, 25%(*w*/*v*) polyethylene glycol 3350, 20%(*v*/*v*) glycerol]. Two sets of SAD data (Zn1 and Zn2 in Supplementary Table S1^1^) were collected from two different crystals at 100 K using an X-ray wavelength of 1.2823 Å on beamline 7A of Pohang Light Source. Raw data were processed and scaled using *HKL*-2000. Anomalous difference maps were calculated using *FFT* from the *CCP*4 suite (Read & Schierbeek, 1988 ▶; Winn *et al.*, 2011 ▶). The two Zn-substituted Csd4 structures are essentially identical to the Csd4–*m*DAP structure in their overall structures. They contain different sets of Zn^2+^ and Ca^2+^ ions, possibly owing to different experimental conditions of EDTA treatment and Zn^2+^ substitution. The Zn1 model contains eight Zn^2+^ ions and one Ca^2+^ ion; the Ca^2+^ ion in the active site and the Ca^2+^ ion that stabilizes the β11–α7 loop are replaced by Zn^2+^. The Zn2 model has two Zn^2+^ and two Ca^2+^ ions; the Ca^2+^ ion bound to the C-terminal Ig-like domain is replaced by Zn^2+^. Other Zn^2+^ ions present in the Zn1 and Zn2 models are likely to be owing to nonspecific binding of Zn^2+^ ions by Csd4. Nonspecific binding of Zn^2+^ ions by proteins was exploited in solving the phase problem by Zn anomalous scattering (Cha *et al.*, 2012 ▶).

### 2.4. Mass spectrometry   

Mass spectra were acquired using a matrix-assisted laser desorption/ionization quadrupole ion-trap time-of-flight mass spectrometer (MALDI-QIT-TOF MS, AXIMA QIT; Shimadzu/Kratos, Manchester, England) equipped with a nitrogen laser (337 nm, 3 ns pulse width, maximum pulse rate 10 Hz). Mass spectra were obtained in the mass range (in Da) from 500 to 2000 *m*/*z* in positive-ion mode. Helium was used for trapping and cooling ions in the ion source. The pressure in the trap was held at 0.53 Pa. Each MS spectrum was constituted of an average of 200 profiles. All spectra were externally calibrated with bradykinin (*m*/*z* 757.3992), angiotensin II (*m*/*z* 1046.5418), angiotensin I (*m*/*z* 1296.6848), Glu-fibrinopeptide B (*m*/*z* 1570.6768) and *N*-acetyl renin substrate (*m*/*z* 1800.9432) in TOFMix (Shimadzu, Japan). Acquisition and data processing were controlled by the *LaunchPad* software. The analyte, NAM-tripeptide at 5 m*M*, was incubated with the recombinant Csd4 protein (5 µ*M*) in the presence or absence of 2.5 m*M* metal ion (Zn^2+^, Ca^2+^ or Mn^2+^) dissolved in 10 m*M* Tris–HCl pH 7.9. A sample solution (1 µl) was mixed on the target with a fresh saturated matrix solution of 2,5-dihydroxybenzoic acid dissolved in 0.1%(*v*/*v*) trifluoroacetic acid and 50%(*v*/*v*) acetonitrile. For sample deposition, a 384-position stainless-steel sample plate was used.

### 2.5. Surface plasmon resonance   

Direct binding of Csd5 to Csd4 was assessed using a Reichert SR7500 surface plasmon resonance (SPR) dual-channel instrument (Reichert, Depew, New York, USA). Purified Csd4 in 20 m*M* sodium acetate pH 5.5 was immobilized using standard amino coupling at 10 µl min^−1^ on a carboxymethyl dextran hydrogel surface sensor chip (Reichert, Depew, New York, USA) until saturation was achieved. The running buffer *C* used in all SPR experiments was 10 m*M* HEPES pH 7.5, 100 m*M* sodium chloride. SPR experiments were performed at 25°C. Csd5 (0.3, 0.5, 1.0 and 2.0 µ*M*) was injected over the Csd4 chip at 30 µl min^−1^ for 4 min for association analyses. Subsequently, the running buffer was flowed over the chip for an additional 4 min (30 µl min^−1^) for molecular-dissociation analyses. To remove metal ions that might be intrinsically bound to Csd4, the Csd4-immobilized chip was treated with running buffer *C* containing 20 m*M* EDTA, the running buffer *C* was flowed over the chip and Csd5 was injected over the Csd4 chip, showing no binding signal. Subsequently, running buffer *C* containing 5 m*M* calcium chloride was passed over the immobilized Csd4, which recovered the Csd4–Csd5 binding. Regeneration of the chip was carried out using 20 m*M* sodium hydroxide solution. Binding was detected as a change in the refractive index at the surface of the chip as measured in response units (RU). A reference flow cell was used to record the response by bovine serum albumin (BSA) as a positive control, and the response by BSA was subtracted from each sample. SPR data were fitted using the *Scrubber*2 software.

### 2.6. Fluorescence polarization binding assay   

The FITC-labelled dipeptide (l-Ala-d-Glu), synthesized by Anygen Co. Ltd, Republic of Korea, was dissolved in and diluted with the binding buffer (10 m*M* HEPES pH 7.5, 100 m*M* sodium chloride) to 9.2 n*M* equally in each 100 µl reaction well. The recombinant Csd5 was also serially diluted in the binding buffer and mixed into each reaction well at concentrations ranging from 17.0 n*M* to 17.4 µ*M*. To detect the change in light polarization of the FITC-labelled dipeptide, fluorescence measurements were made in a 96-well format plate reader (Molecular Devices) with excitation and emission wavelengths of 485 and 538 nm, respectively. A nonlinear graph of dipeptide concentration-dependent polarization was produced using *Prism* 6 (GraphPad).

### 2.7. Accession codes   

The coordinates and structure factors have been deposited in the Protein Data Bank under accession codes 4q6m, 4q6n and 4q6o for the Csd4-unbound, Csd4–muramyltripeptide and Csd4–*m*DAP structures, respectively, and 4q6p and 4q6q for the Zn-bound forms of Csd4.

## 3. Results and discussion   

### 3.1. Structure determination of *H. pylori* Csd4   

**Figure 1:**
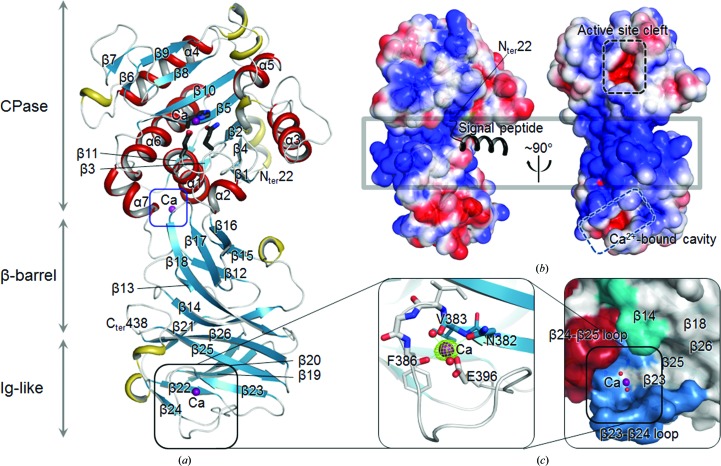
Overall structure of *H. pylori* Csd4. (*a*) Ribbon diagram of the Csd4-unbound structure. β-Strands, α-helices, 3_10_-helices and loops are shown in cyan, red, yellow and grey, respectively. Three calcium ions are shown as purple spheres. The metal-binding residues in the N-terminal CPase domain are shown as stick models. The blue and black boxes indicate the previously unknown Ca^2+^-binding sites of the CPase domain and the Ca^2+^-binding motif of the C-­terminal Ig-like domain. The secondary-structure elements were defined by *DSSP* (Kabsch & Sander, 1983 ▶). (*b*) Electrostatic surface diagram of Csd4-unbound. The signal peptide is modelled as a black ribbon diagram. The black and cyan dotted boxes indicate the active-site cleft of the CPase domain and the Ca^2+^-binding channel of the Ig-like domain. The grey box indicates the highly positively charged surface of the central β-barrel domain. Electrostatic potential at the molecular surface was calculated using *APBS* (Baker *et al.*, 2001 ▶). (*c*) Ribbon diagram of the Ca^2+^-binding motif (left) and the surface representation of the Ca^2+^-bound channel (right) in the Ig-like domain. The OMIT *mF*_o_ − *DF*_c_ map (contoured at 10σ) for the calcium ion is coloured green. The Ca^2+^-binding residues are shown as stick models. The calcium ion and the bound waters are shown as purple and red spheres, respectively. All figures representing the protein structure were drawn using *PyMOL* (v.1.3r1; Schrödinger).

We have solved the crystal structure of *H. pylori* Csd4 using Se-SAD data (Table 1 ▶). Subsequently, we have refined three models of *H. pylori* Csd4: ligand-free (Csd4-unbound), substrate-bound (Csd4–muramyltripeptide) and product-bound (Csd4–*m*DAP) structures (Table 1 ▶). All of our Csd4 crystals contain one Csd4 monomer in the asymmetric unit. These models account for all of the amino-acid residues of the recombinant Csd4 (residues Met22–Val438) except for the N-terminal affinity tag (Fig. 1 ▶ *a*). The peptide portion of the bound NAM-tripeptide and *m*DAP as well as three metal ions bound to Csd4 are well defined by the electron density. Since the crystallization reservoir solution contained calcium chloride, we have assigned the three metal ions as Ca^2+^. Metallopeptidases utilize a catalytic Zn^2+^ bound to the active site. Therefore, our crystallized enzyme with a Ca^2+^ ion in the active site is a noncompetent variant. Further anomalous scattering experiments confirmed that these calcium ions could be substituted by zinc ions (Supplementary Fig. S1 and Supplementary Table S1).

The three models are highly similar to each other, with r.m.s. deviations of 0.17–0.38 Å for pairwise comparisons over 417 C^α^-atom pairs. Interestingly, large C^α^ deviations occur in the β9–α5 loop, with values of 4.2 and 3.7 Å for Asp200 and Glu202, respectively (Supplementary Fig. S2). This region is well defined by the electron density in all three models and the observed large structural difference is induced by ligand binding, as discussed in more detail below. Csd4 exists as monomers in the crystals, as the largest surface areas buried at the interface between two neighbouring monomers within the crystals are 502, 467 and 481 Å^2^ per monomer (2.7, 2.5 and 2.6% of the monomer surface area) for the Csd4-unbound, Csd4–muramyltripeptide and Csd4–*m*DAP structures, respectively. This conclusion from the crystallo­graphic data is also supported by the results of size-exclusion chromatography of the protein in solution.

### 3.2. Three-domain structure of *H. pylori* Csd4   

*H. pylori* Csd4 consists of three domains: an N-terminal CPase domain (Met22–Asn267), a central β-barrel domain (Asp268–Glu340) with a novel fold and a C-terminal immuno­globulin (Ig)-like domain (Phe341–Val438) (Fig. 1 ▶ *a*). The three domains are arranged in a serial order to form a curved overall structure (Fig. 1 ▶ *b*, left panel). When we performed *DALI* searches (Holm & Rosenström, 2010 ▶) with individual domains, only the N-terminal CPase domain showed significant structural similarity to other known proteins, as described below. A *DALI* search with the whole chain of Csd4 gave essentially the same result as with the N-terminal CPase domain only.

The N-terminal CPase domain with a CPase fold has a central seven-stranded β-sheet (β1↑–β4↓–β2↓–β5↓–β10↓–β8↑–β9↑), which is highly twisted and spans the entire CPase domain from top to bottom (Fig. 1 ▶ *a*). This gives rise to a concave face, which accommodates helices α3 and α5 and a short β-sheet (β6↑–β7↑) as well as the active-site cleft containing a metal ion (Figs. 1 ▶ *a* and 2 ▶ *a*). The convex side of the sheet harbours four helices (α1, α4, α6 and α7) and a short β-sheet (β3↑-β11↑) which contributes to another metal-binding site, thereby stabilizing the β11–α7 loop (indicated by a blue box in Fig. 1 ▶ *a* and Supplementary Fig. S1*b*). The N-terminal domain of Csd4 has a canonical metallo-CPase-like architecture. It shows structural similarity to members of the CPase B protein family such as the activated porcine pancreatic carboxypeptidase B (CPase B) as a surrogate of human TAFIa (thrombin-activatable fibrinolysis inhibitor; Bunnage *et al.*, 2007 ▶; PDB entry 2jew; *Z*-score of 18.6, r.m.s. deviation of 2.2 Å and sequence identity of 15% for 185 equivalent C^α^ positions) and human CPase A4 (Pallarès *et al.*, 2005 ▶; PDB entry 2bo9; *Z*-score of 17.3, r.m.s. deviation of 2.5 Å and sequence identity of 14% for 187 equivalent C^α^ positions).

The central β-barrel domain appears to have a novel fold, because the highest *Z*-score was only 3.3 with RAS-related C3 botulinum toxin substrate 1 (Grizot *et al.*, 2001 ▶; PDB entry 1hh4, chain *D*). It comprises two antiparallel β-sheets: a major twisted sheet (β16↓–β17↑–β18↓–β14↓) and a minor sheet (β15↑–β12↓–β13↓). Interestingly, the central β-barrel domain has a highly positively charged surface (shown by a grey box in Fig. 1 ▶ *b*), contributing to a high overall isoelectric point (pI) of the protein (theoretically 9.0). The theoretical pI of the central β-barrel domain is 10.0, while those of the N- and C-terminal domains are 6.7 and 8.1, respectively.

The overall fold of the C-terminal Ig-like domain containing two β-sheets (β20↑–β19↓–β22↑–β24↓ and β21↓–β26↓–β25↑-β23↓) resembles an Ig fold, even though the structural similarity is rather low. The highest similarity was detected in the Ig-like Y_Y_Y domain of the *Bacteroides thetaiotaomicron* histidine kinase (Lowe *et al.*, 2012 ▶; PDB entry 4a2l; *Z*-score of 5.2, r.m.s. deviation of 2.9 Å for 78 equivalent C^α^ positions and sequence identity of 13%). However, the key residues (three Tyr residues) of the Y_Y_Y domain are not well conserved in the C-terminal domain of Csd4; the equivalent residues are Arg380, Asn382 and Tyr419. The C-terminal domain of Csd4 might be a member of a new subfamily of the Ig-like fold superfamily (Pfam CL0159). The Ig-like domain of Csd4 is decorated with several winding loops, including the β2–β25 loop, and two additional 3_10_-helices. Unexpectedly, these structural elements form a Ca^2+^-binding channel (Fig. 1 ▶ *c*, right panel). The OMIT electron-density map of Csd4-unbound clearly indicates that a calcium ion is bound to the C-terminal Ig-like domain and is coordinated by six ligand atoms, *i.e.* the side-chain atoms of the highly conserved Asn382 (located on β23) and Glu396 (located on the β23–β24 loop) and the main-chain carbonyl O atoms of Val383 and Phe386 (Fig. 1 ▶ *c*, left panel; Supplementary Fig. S1*c*). The surface of the calcium ion-bound channel in the C-terminal Ig-like domain (Fig. 1 ▶ *c*, right panel) is lined with the β23–β24 and β24–β25 loops and strands β23 and β25. It is further contributed to by the long side chains of residues Asn291, His292, Phe337 and Ile339 from strands β14 and β18 of the central β-barrel domain (Fig. 1 ▶ *c*, right panel).

### 3.3. Unique features of the Csd4 d,l-carboxypeptidase domain   

**Figure 2:**
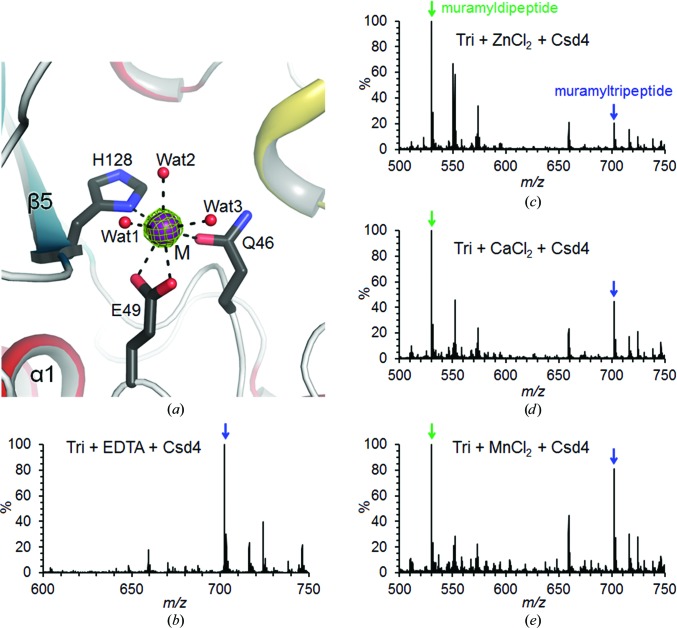
Metal-dependency of *H. pylori* Csd4 as a carboxypeptidase. (*a*) Ribbon diagram of the active site in the Csd4-unbound structure coloured as in Fig. 1 ▶(*a*). The OMIT *mF*_o_ − *DF*_c_ map (contoured at 5σ) for the calcium ion is coloured green. (*b*–*e*) Mass spectra of muramylpeptides after the reaction catalyzed by Csd4 in the presence of EDTA (*b*) or in the presence of Zn^2+^ (*c*), Ca^2+^ (*d*) or Mn^2+^ (*e*). Green and blue arrows indicate the peaks corresponding to the muramyldipeptide and muramyltripeptide (Tri), respectively.

The N-terminal CPase domain of Csd4 has a highly twisted β-sheet, forming an active-site cleft on a concave face (indicated by a black dotted box in Fig. 1 ▶ *b*). The access to the active-site cleft is reminiscent of a funnel, the rim of which is formed by four surface loops (α2–α3, β2–α1, β7–β8 and β9–α5). In the Csd4-unbound structure, which represents the substrate-free state, a metal ion is bound to the active-site cleft with a broad entrance (Fig. 2 ▶ *a*). It was reported that Csd4 requires divalent metal ions to cleave the peptide bond (d-Glu^2^-*m*DAP^3^) of a disaccharide-tripeptide (NAG-NAM-l-Ala^1^-γ-d-Glu^2^-*m*DAP^3^; Sycuro *et al.*, 2012 ▶). We tested the CPase activity of Csd4 with the synthetic NAM-tripeptide as the substrate in the presence of Zn^2+^, Ca^2+^ and Mn^2+^. Based on the mass analysis, we found that Csd4 trims the NAM-tripeptide to the NAM-dipeptide (NAM-l-Ala^1^-γ-d-Glu^2^) in the presence of any one of these metal ions, releasing *m*DAP (Figs. 2 ▶ *c*–2 ▶ *e*). The highest activity was observed with Zn^2+^. No such reaction was observed in the presence of EDTA (Fig. 2 ▶ *b*). Clear electron density is present at the metal-binding site of the CPase domain in our Csd4-unbound (Fig. 2 ▶ *a*), Csd4–muramyltripeptide and Csd4–*m*DAP structures. We interpreted it as Ca^2+^ because the crystallization condition contained a high concentration of Ca^2+^. We have confirmed this interpretation by collecting anomalous data at the Zn absorption edge (1.2823 Å). The anomalous difference map clearly showed that this metal ion is not Zn^2+^ in the Csd4-unbound crystal. Further anomalous scattering experiments on the crystal that was soaked in Zn^2+^ after EDTA treatment indicated that this metal ion can be replaced by Zn^2+^ (Zn1 data set in Table S1; Supplementary Fig. S1*a*). These results support that Csd4 employs a catalytic zinc ion in the active site like many other carboxy-metallopeptidases. Therefore, our crystallized enzyme with a calcium ion in the active site is a noncompetent variant.

The N-terminal CPase domain of Csd4 shows structural similarity to M14 metallopeptidases such as pig CPase B (PDB entry 2jew; Bunnage *et al.*, 2007 ▶) and human CPase A4 (PDB entry 2bo9; Pallarès *et al.*, 2005 ▶), both of which are members of the M14A subfamily. To date, four subfamilies (M14A–M14D) have been identified among the M14 metallopeptidase family (MEROPS peptidase database; Rawlings *et al.*, 2014 ▶). Similarly to both human CPase B and CPase A4, Csd4 cleaves the C-terminal residue. However, despite its structural similarity to M14 metallopeptidases, Csd4 appears to be unique among M14 family members regarding substrate specificity and active-site residues, including the metal-binding motif, as discussed below in detail. We therefore propose that Csd4 belongs to a new metallo-CPase M14 subfamily distinct from the known M14 subfamilies M14A–M14D.

**Figure 3:**
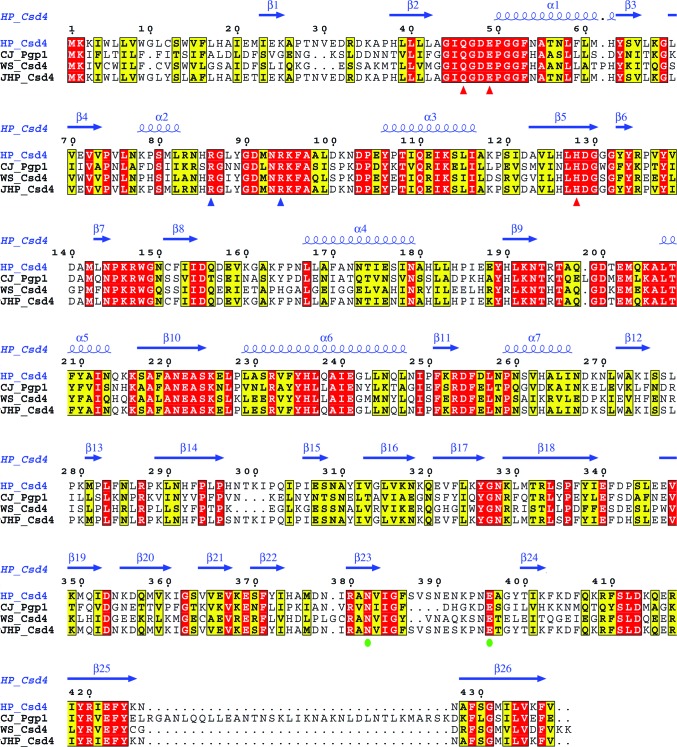
Sequence alignment of four Csd4 homologues. Sequence alignment of Csd4 from *H. pylori* strain 26695 (HP_Csd4; SWISS-PROT accession code O25708), Pgp1 from *Campylobacter jejuni* (CJ_Pgp1; A1W0W1), Csd4 from *Wolinella succinogenes* (WS_Csd4; Q7MSQ3) and Csd4 from *H. pylori* strain J99 (JHP_Csd4; Q9ZM72) was performed and presented using *ClustalX* (Larkin *et al.*, 2007 ▶) and *ESPript* (Gouet *et al.*, 2003 ▶; http://espript.ibcp.fr). Red and blue triangles indicate the metal-binding site motif (Gln46, Glu49 and His128) and the two key Arg residues (Arg86 and Arg94) showing conformational changes in the active site of the CPase domain. Green circles indicate the Ca^2+^-binding motif in the C-terminal Ig-like domain.

Firstly, Csd4 is active without proteolytic processing owing to the lack of a pro-domain for a zymogenic form (Figs. 2 ▶ *c*–2 ▶ *e*), whereas both pig CPase B and human CPase A4 require proteolytic cleavage of their precursor forms for activation (Bunnage *et al.*, 2007 ▶; Pallarès *et al.*, 2005 ▶). The substrate specificity of Csd4 is also different from those of the known M14 subfamilies: M14A subfamily CPases favour residues with aromatic or branched side chains and M14B and M14D subfamily CPases prefer basic amino acids. While Csd4 cleaves the peptide bond of d-Glu^2^-*m*DAP^3^ derived from peptidoglycan with concurrent recognition of its C-terminus, M14C family members carry out hydrolysis at the same cleavage site of peptidoglycan but they behave as endopeptidases. In addition, the overall three-domain architecture of *H. pylori* Csd4, consisting of an N-terminal CPase domain, a central β-barrel domain and a C-terminal Ig-like domain, is conserved in Csd4 homologues from other bacteria (Fig. 3 ▶), but is absent in other CPases with known structures. Interestingly, Csd4 homologues are mainly found in spiral-shaped bacteria (Sycuro *et al.*, 2012 ▶; Frirdich *et al.*, 2012 ▶).

Next, the CPase domain (Met22–Asn267) of *H. pylori* Csd4 shows relatively large r.m.s. deviations (2.2 Å for 185 equivalent C^α^ positions and 2.5 Å for 187 equivalent C^α^ positions, respectively) and low sequence conservation (sequence identities of 15 and 14%, respectively) when compared with pig CPase B and human CPase A4. The central β-sheet of the Csd4 CPase domain has essentially the same topology as in other CPases. However, it consists of only seven strands, lacking a β-strand equivalent to β0′′ of pig CPase B and human CPase A4. In addition, an extra α-helix (α0′′) is present at the N-terminus of pig CPase B and human CPase A4 (Supplementary Fig. S3). In the Csd4 structure this α-helix is missing and is substituted by the central β-barrel domain (Supplementary Fig. S3). Another α-helix of pig CPase B and human CPase A4 is replaced by a mini two-stranded β-sheet (β3 and β11) in Csd4. Large structural differences also exist in the loops around the active-site cleft. The four loops (α2–α3, β5–β8, β8–α4 and β9–α5) contribute to the unique shape of the Csd4 active-site cleft and determine its substrate specificity (Supplementary Fig. S3). The Csd4 β5–β8 loop contains an extra two-stranded β-sheet formed by β6–β7 that is absent in other CPases.

Finally, the metal-binding motif in the CPase domain of Csd4 differs from those in other CPases. In the Csd4-unbound, Csd4–muramyltripeptide and Csd4–*m*DAP structures a calcium ion is coordinated in an octahedral fashion by Gln46, Glu49 and His128 from the metal-binding motif (**Q** *xx* **E** *x*_*n*_ **H**), which is strictly conserved in Csd4 homologues (Fig. 3 ▶), and three water molecules, with metal–ligand atom distances of 2.3–2.6 Å (Fig. 2 ▶ *a*). *x* stands for any amino acid and the strictly conserved residue is in bold. In contrast, most other known M14 metallo-CPases have a different metal-binding motif, **H** *xx* **E** *x*_*n*_ **H** (Supplementary Fig. S3).

### 3.4. The Csd4–muramyltripeptide and Csd4–*m*DAP structures reveal the substrate-bound and product-bound states   

**Figure 4:**
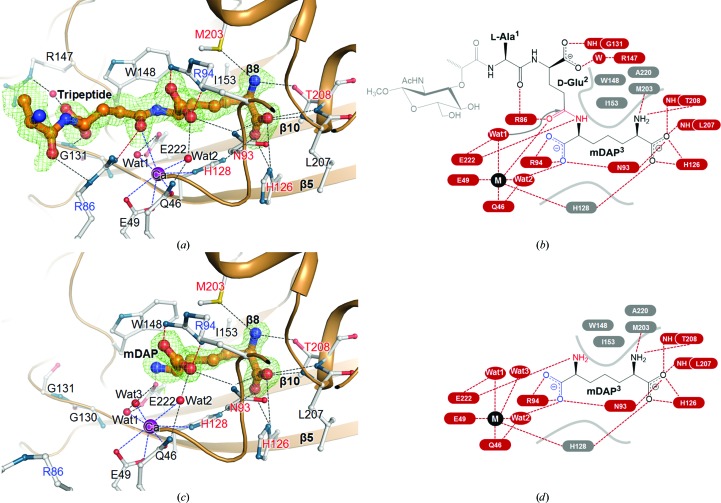
Ligand interactions in the Csd4–muramyltripeptide and Csd4–*m*DAP structures. (*a*, *c*) Ribbon diagrams of the active site in the Csd4–muramyltripeptide (*a*) and Csd4–*m*DAP (*c*) structures. The tripeptide and *m*DAP are shown as stick models. Since the sugar moiety of the NAM-tripeptide was invisible, only the tripeptide portion has been modelled into the electron-density map. Residues interacting with the tripeptide or *m*DAP are shown as stick models. The OMIT *mF*_o_ − *DF*_c_ maps for the tripeptide (contoured at 1.5σ) and *m*DAP (contoured at 2.5σ) are coloured green. The red and blue dotted lines indicate the interactions of the tripeptide or *m*DAP with the metal ion and two key Arg residues (Arg86 and Arg94), respectively. (*b*, *d*) Schematic diagrams of interactions of the bound muramyltripeptide (*b*) and *m*DAP (*d*) with Csd4. The red and grey labels represent electrostatic and hydrophobic residues interacting with these ligands, respectively.

To understand the structural basis for the specific inter­actions of Csd4 with the peptidoglycan-derived muramyltripeptide substrate and the product *m*DAP, we solved the Csd4–muramyltripeptide and Csd4–*m*DAP structures. *m*DAP is the C-terminal residue of the substrate muramyltripeptide and is one of the products produced by the reaction catalyzed by Csd4. Therefore, our Csd4–muramyltripeptide and Csd4–*m*DAP structures represent the substrate-bound and product-bound states prior to and after the cleavage reaction by Csd4, respectively. Clear electron densities were observed both for l-Ala^1^-γ-d-Glu^2^-*m*DAP^3^ in the Csd4–muramyltripeptide structure and for *m*DAP in the Csd4–*m*DAP structure (Fig. 4 ▶). In both structures, extensive interactions exist between *m*DAP and Csd4. They are highly conserved between the two structures, highlighting the importance of these interactions in determining substrate specificity.

**Figure 5:**
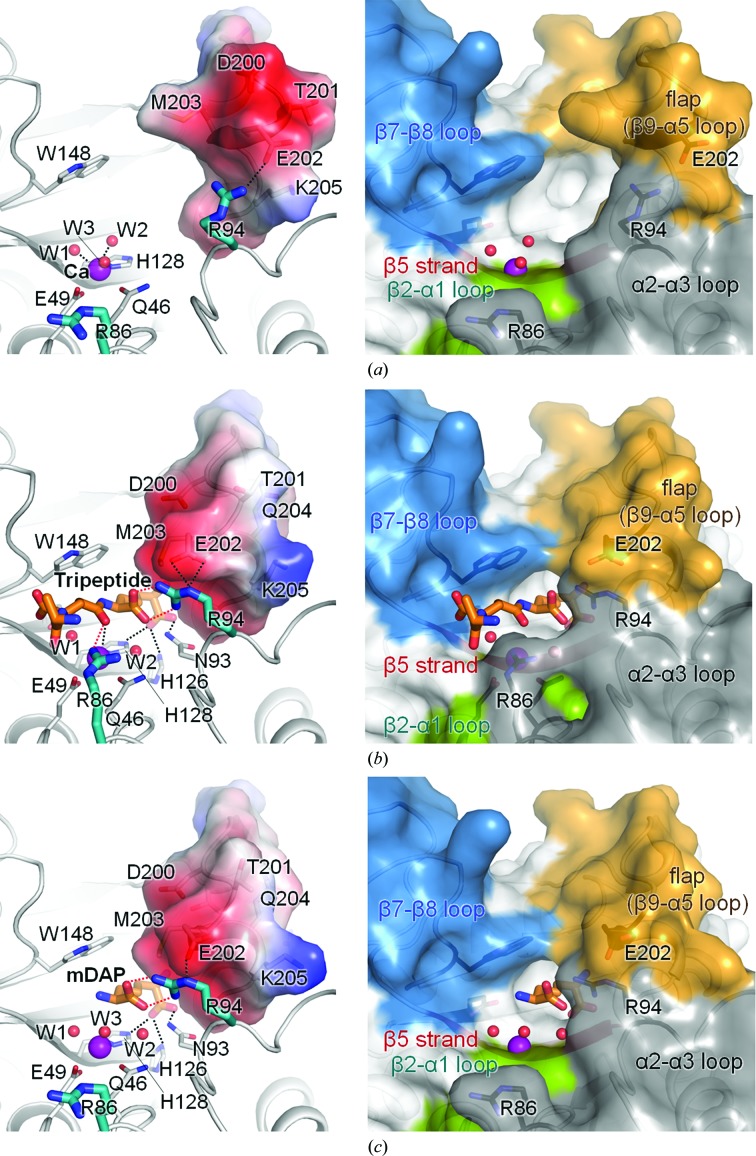
Open and closed conformations of the flap. Electrostatic surface diagrams of the mobile flap (left panels) and surface diagrams showing the active-site cleft (right panels) in the Csd4-unbound (*a*), Csd4–muramyltripeptide (*b*) and Csd4–*m*DAP (*c*) structures.

In the Csd4–muramyltripeptide structure, the bulk of the interactions are centred around the scissile peptide bond, the *m*DAP moiety and the active-site metal ion (Figs. 4 ▶ *a* and 4 ▶ *b*). In this structure, the linear tripeptide part of the substrate lies antiparallel to the β5 strand of Csd4 (Fig. 4 ▶ *a*) and is surrounded by four loops (β2–α1, α2–α3, β7–β8 and β9–α5; Fig. 5 ▶ *b*). The sugar moiety is not well defined in the electron-density map, likely because it protrudes from the active site and does not make a specific interaction with the active-site residues. While the l-Ala^1^-d-Glu^2^ segment of the peptide is largely exposed to the solvent, it is well defined by the electron density as it makes specific interactions with Csd4. The *m*DAP^3^ part sits deep in the active-site cleft and is enclosed by the loops (Fig. 5 ▶ *b* and 5 ▶ *c*), thus making extensive inter­actions with Csd4. The main-chain carbonyl O atom of l-Ala^1^ makes a polar interaction with the side chain of Arg86 (Figs. 4 ▶ *a* and 4 ▶ *b*). The aliphatic portions of d-Glu^2^ and *m*DAP^3^ make nonpolar interactions with hydrophobic parts of the Trp148, Ile153, Met203, Ala220 and His128 side chains (Figs. 4 ▶ *a* and 4 ▶ *b*). The scissile carbonyl O atom of d-Glu^2^ interacts with the side chain of Arg86. The carboxylate group of d-Glu^2^ is bonded to Gly131^N^ and a water molecule, which is hydrogen-bonded to Arg147. Notably, the terminal *m*DAP^3^ is involved in tight interactions with Csd4. The side-chain amine N atom of *m*DAP^3^ interacts with Met203^δ^ and Thr208^γ^. The side-chain carboxylate O atoms of *m*DAP^3^ make contacts with Asn93^δ2^, His126^δ1^, His128^∊2^, Leu207^N^ and Thr208^N^.

The *m*DAP-dependency of selectivity is revealed in the Csd4–*m*DAP structure as well as in the Csd4–muramyltripeptide structure (Figs. 4 ▶ *c* and 4 ▶ *d*). The binding of *m*DAP alone induces ligand interactions similar to the case of Csd4–muramyltripeptide (Fig. 4 ▶). The shape of the active-site cleft of Csd4–*m*DAP is nearly identical to that of Csd4–muramyltripeptide; *m*DAP of Csd4–*m*DAP is well ensconced in the cleft (Figs. 4 ▶ *b* and 4 ▶ *c*), suggesting that the presence or absence of the *m*DAP segment is one of the key factors in determining the suitable shape of the active-site cleft for catalysis and is an important factor in substrate selectivity.

The Csd4–muramyltripeptide structure shows that the tripeptide moiety and the metal ion are suitably predisposed for interaction during the course of catalysis. The metal ion is coordinated by Gln46^∊1^, Glu49^∊1,∊2^ and His128^δ1^ and two water molecules (Wat1 and Wat2). However, a third water molecule (Wat3), one of the six metal-binding ligands in the Csd4-unbound structure, is substituted by the carbonyl O atom of the scissile peptide bond between d-Glu^2^ and *m*DAP^3^ in the Csd4–muramyltripeptide structure, thereby enabling a direct interaction with the metal ion to polarize the peptide carbonyl bond. The carbonyl O atom of the scissile peptide bond is also hydrogen-bonded to Arg86^η1^ with a distance of 2.8 Å to stabilize the transient oxyanion species of the polarized carbonyl bond (Fig. 4 ▶). This species is the product of the addition of Wat1 to the carbonyl carbon, which would be promoted by Glu222 as a general base. Furthermore, the scissile amide N atom interacts with the carboxyl group of Glu222^∊1^, rendering it susceptible to nucleophilic attack. The water molecule Wat1, interacting with both Glu222^∊1,∊2^ and the metal ion, is well positioned for nucleophilic attack at the carbonyl C atom of the scissile bond with a distance of 3.2 Å (Fig. 4 ▶). In addition, Csd4 is set to recognize the C-terminal carboxylate of *m*DAP^3^. The Csd4–muramyltripeptide structure shows that Arg94 is the key residue for recognition of the C-terminal *m*DAP residue of the substrate. The free C-terminal carboxylate group of *m*DAP^3^ is salt-bridged to Arg94^η1^ and Arg94^η2^ in a bidentate manner, accounting for the specificity of Csd4 as a ‘carboxy’ peptidase. The C-terminal carboxylate group is also bound to Asn93^δ2^ and the metal ion *via* the water Wat2, demonstrating that the metal ion serves to hold key elements for catalyzing the reaction.

### 3.5. Ligand binding modulates the conformation of the CPase domain   

Among the three structures of Csd4, the β9–α5 loop region (residues Thr196–Ala206) containing two 3_10_-helices exhibits the largest structural variation (Supplementary Fig. S2). This region is moved towards the active site in the Csd4–muramyltripeptide and Csd4–*m*DAP structures when we compare them with Csd4-unbound (Fig. 5 ▶, left panels). This flexible region works as a flap and undergoes a conformational change upon binding of the substrate or the product. In the substrate-free state of Csd4-unbound, the flap is positioned away from the active site (Fig. 5 ▶ *a*). Upon binding of the muramyl­tripeptide, it moves closer to the active site and its surface shape and electrostatic potential are altered (Fig. 5 ▶ *b*). As shown in Fig. 5( ▶ *b*), the flap forms the edge of the active-site cleft and is responsible for recognition of the *m*DAP portion of the muramyltripeptide. In the Csd4–*m*DAP structure the flap has a similar conformation as in the Csd4–muramyltripeptide structure, indicating that the conformation of the mobile flap is primarily influenced by the *m*DAP moiety.

Depending on the ligand binding, we observe interesting differences in the orientation of the side chains of two key arginine residues on the α2–α3 loop, *i.e.* Arg86 and Arg94 (Fig. 5 ▶). Their side chains are well defined by a clear electron density. In the Csd4–muramyltripeptide structure, the side chain of Arg86 is directed towards the active site to interact with the O atom of the scissile peptide bond of the bound muramyltripeptide (Fig. 5 ▶ *b*). In comparison, it points away from the active site in the Csd4-unbound and Csd4–*m*DAP structures (Figs. 5 ▶ *a* and 5 ▶ *c*). Compared with Csd4-unbound and Csd4–*m*DAP, the N^η^ atom of Arg86 in Csd4–muramyltripeptide is moved by about 8 Å so that Arg86 is recruited for binding d-Glu^2^-*m*DAP^3^ within the substrate. Arg94, which is positioned between the active site and the mobile flap, plays an interesting role in the flap movement by interacting with both the free C-terminal carboxylate group of the *m*DAP part and Glu202 of the flap. The side chain of Arg94 interacts with the flap *via* a strong salt bridge with Glu202 in all three structures. It points towards the active site in both the Csd4–muramyltripeptide and Csd4–*m*DAP structures, whereas it points away from the active site in the Csd4-unbound structure (Fig. 5 ▶). In other words, Arg94 pulls the flap towards the active site *via* a strong salt bridge with Glu202 in both the Csd4–muramyltri­peptide and Csd4–*m*DAP structures when its side chain recognizes the free C-terminal carboxylate group of *m*DAP. Furthermore, this structural change induces an additional interaction between the side-chain amine group of *m*DAP and Met203 (Fig. 4 ▶). Therefore, we suggest that Arg94 is the key determinant of selectivity in the ‘carboxy’-peptidase activity of Csd4.

The conformations of the flap (β9–α5 loop) and the two key Arg residues (Arg86 and Arg94) on the α2–α3 loop affect the shape and size of the active site, as they form the wall of the active-site cleft. Loops forming the surface of the active site define the front and the bottom of the active site and the specificity of the active-site pocket (Fig. 5 ▶, right panels). The Csd4-unbound structure reveals an open active-site cleft in the substrate-free state, thus exposing the metal ion and the inside of the cleft to the bulk solvent (Fig. 5 ▶ *a*). In this structure, the flap is in the open conformation and the two Arg residues point towards the outside of the active site, predisposing the enzyme to binding of the substrate. In the Csd4–muramyltripeptide structure the flap is in the closed conformation and the tripeptide moiety of the substrate fits nicely into the active site, thus interacting extensively with the loops around the active site (Fig. 5 ▶ *b*). In the Csd4–*m*DAP structure the flap is also in the closed conformation and the inner side of the active-site cleft is occupied by *m*DAP and is covered by the flap, while the outer side of the cleft is open towards the surface, exposing the metal site to the bulk solvent (Fig. 5 ▶ *c*).

Taken together, these findings enable us to propose a catalytic mechanism for *H. pylori* Csd4 as a d,l-CPase. After access through an open entrance to the active-site pocket (Fig. 5 ▶ *a*), the substrate carbonyl O atom replaces a metal-bound water molecule (Wat3) to interact with the metal ion directly. By abstracting a proton from a water molecule (Wat1), the active-site Glu222 promotes the water for nucleophilic attack of the sessile carbonyl, en route to formation of the reactive tetrahedral species. Glu222 shuttles the proton to the departing amide N atom of the sissile amide bond. The metal ion serves to stabilize Wat1 and the scissile carbonyl of the substrate. Arg86 is then recruited to interact with the scissile carbonyl O atom to both position the carbonyl O atom and to stabilize the tetrahedral species. In addition, Arg94 fixes the position of the C-terminal carboxylate of the bound substrate, inducing the flap movement to cover *m*DAP^3^. Protonation of the amide N atom is the committed step in bond scission leading to the two products.

### 3.6. Csd4 interacts physically with Csd5   

**Figure 6:**
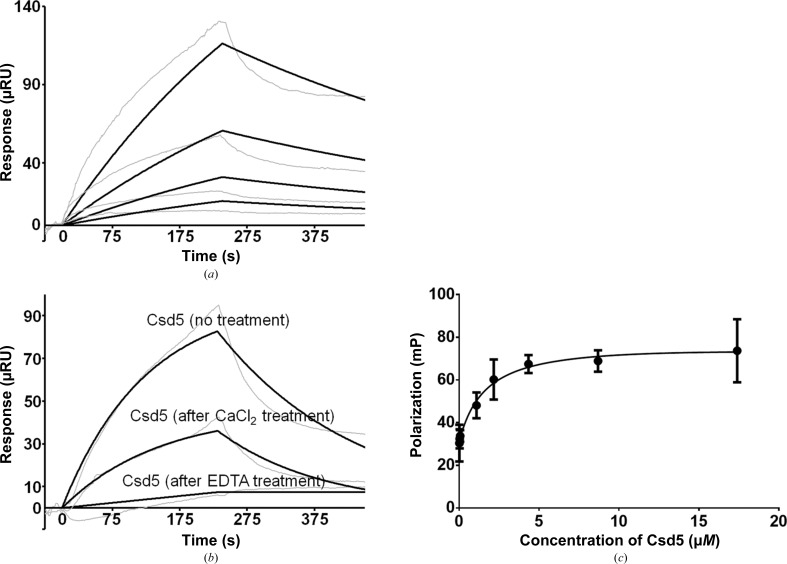
Biophysical studies of the Csd4–Csd5 or the Csd5–dipeptide interaction in solution. (*a*) SPR experiments with immobilized Csd4 and Csd5 as an analyte at different concentrations (0.25, 0.50, 1.00 and 2.00 µ*M*) are shown as grey-coloured traces. Black traces show the corresponding binding-model curves. (*b*) At the same concentration of Csd5 (2.00 µ*M*), the interaction between Csd4 and Csd5 (‘no treatment’) was lost upon treatment of the Csd4-immobilized chip with 20 m*M* EDTA to remove intrinsic metal ions (‘after EDTA treatment’). Retreatment of the EDTA-treated Csd4-immobilized chip with 5 m*M* calcium chloride recovered the binding between two proteins (‘after CaCl_2_ treatment’). All sensorgrams were obtained by subtracting the nonspecific binding of the analyte to the BSA-immobilized chip. (*c*) Fluorescence polarization binding assay using an FITC-labelled dipeptide against increasing concentrations of Csd5.

Previously, the *csd4csd5* double mutant was reported to resemble the *csd4* mutant both in global peptidoglycan changes and by having a straighter shape than the *csd5* mutant, suggesting that Csd4 acts upstream of Csd5 (Sycuro *et al.*, 2012 ▶). This suggests that Csd4 may interact physically with Csd5, another cell-shape determinant. To date, no such interaction between Csd4 and Csd5 has been demonstrated. Therefore, we investigated whether Csd4 could interact directly with Csd5. The Csd4–Csd5 interaction was detected by SPR using immobilized Csd4 with Csd5 as the analyte at concentrations between 0.25 and 2.00 µ*M*. The dissociation constant (*K*_d_) was calculated as ∼4.5 µ*M* (Fig. 6 ▶ *a*). This shows that Csd5 can bind saturably to Csd4, which is indicative of an evolved function of the formation of a complex between the two proteins. Interestingly, the observed interaction between Csd4 and Csd5 disappeared upon treatment of the Csd4-immobilized chip with 20 m*M* EDTA to remove intrinsically bound metal ions (Fig. 6 ▶ *b*). Moreover, retreatment of the EDTA-treated Csd4-immobilized chip with 5 m*M* calcium chloride recovered the binding between the two proteins, yielding a similar *K*_d_ value (∼4.3 µ*M*; Fig. 6 ▶ *b*). Therefore, we conclude that Csd4 physically interacts with Csd5 in a Ca^2+^-dependent manner. Upon showing that Csd5 interacts physically with Csd4, we hypothesized that Csd5 may also interact with the peptidoglycan-derived dipeptide which is the product of the enzymatic reaction catalyzed by Csd4. An FITC-labelled dipeptide (FITC-l-Ala-d-Glu) was synthesized and was used to carry out fluorescence polarization experiments. The FITC-labelled dipeptide was found to bind to Csd5 with a *K*_d_ value of ∼1.69 µ*M* (Fig. 6 ▶ *c*). Therefore, we suggest that Csd5 is capable of recognizing both Csd4 and the dipeptide product of Csd4 downstream of the transformation catalyzed by Csd4.

## 4. Conclusions   

The non-invasive Gram-negative pathogen *H. pylori* is recognized by epithelial cells *via* the Nod1 receptor (Viala *et al.*, 2004 ▶). The substrate of Csd4, *m*DAP-containing muramyltripeptide, is an agonist for the cytosolic innate immune receptor Nod1 (Girardin *et al.*, 2003 ▶; Chamaillard *et al.*, 2003 ▶), and thus a decreased amount of muramyltripeptide as a result of catalysis by Csd4 is likely to affect Nod1 activation and ultimately NF-κB activity. Δ*pgp1*, the mutant of the Csd4 homologue in *C. jejuni*, produces a significantly higher interleukin-8 (IL-8) response in human epithelial cells (INT407) than does wild-type *C. jejuni* (Frirdich *et al.*, 2012 ▶; Sycuro *et al.*, 2012 ▶), even though the Δ*csd4* mutant is comparable to wild-type *H. pylori* in pro-inflammatory cytokine induction of gastric epithelial AGS cells (Sycuro *et al.*, 2012 ▶). Therefore, it is suggested that d,l-carboxypeptidation by Csd4 homologues could affect the overall amount of released *m*DAP-containing muramyltripeptides, affect the helical shape and influence immune detection (Frirdich *et al.*, 2012 ▶; Wyckoff *et al.*, 2012 ▶).

In this work, we report the crystal structure of Csd4 in three different states. The N-terminal CPase domain has unique features among members of the M14 metallopeptidase family. Our Csd4–muramyltripeptide and Csd4–*m*DAP structures represent the substrate-bound and product-bound states, showing how the *m*DAP-containing muramyltripeptide could be cleaved to the dipeptide variant by Csd4. In the two complex structures extensive interactions between Csd4 and ligands are centred on the *m*DAP moiety, highlighting the important role of *m*DAP in specific substrate recognition. Moreover, we also show that the shift of the flap (β9–α5 loop) and two key Arg residues (Arg86 and Arg94) upon binding the muramyltripeptide or *m*DAP affects the shape and the size of the active-site cleft.

*H. pylori* Csd4 catalyzes the final step in the generation of the shortest muropeptide from the peptidoglycan, so that its catalytic process and localization should be tightly regulated to control the cell shape, the host immune response and even new peptidoglycan synthesis. *H. pylori* Csd5 has no known enzymatic activity, exerts little effect on the global peptido­glycan composition and is not required for Csd4 activity. Csd4 was suggested to act upstream of Csd5, raising the possibility that Csd5 could directly interact with Csd4 or the muramyl­dipeptide product of the Csd4-catalyzed reaction. Here, we have demonstrated that Csd5 interacts physically with both Csd4 and the dipeptide product of the reaction catalyzed by Csd4. We suggest that Csd5 may play a regulatory role in modulating the function of Csd4. This study reports the first structural insights into the events that are influenced by Csd4 of *H. pylori*, which will serve as a point of departure for deeper understanding of the regulatory events leading to the proper cell shape.

## Supplementary Material

PDB reference: Csd4, Zn-bound form I, 4q6p

PDB reference: Zn-bound form II, 4q6q

PDB reference: apo, 4q6m

PDB reference: tripeptide-bound form, 4q6n

PDB reference: *m*DAP-bound form, 4q6o

Supporting Information.. DOI: 10.1107/S1399004714018732/mh5144sup1.pdf

## Figures and Tables

**Table 1:** Statistics of data collection and refinement Values in parentheses are for the highest resolution shell.

Data set	Csd4-unbound	Csd4muramyltripeptide	Csd4*m*DAP	SeMet (peak)
Data collection				
Beamline source†	PF BL-17A	PLS BL-7A	PLS BL-7A	PLS BL-7A
X-ray wavelength ()	1.0722	0.9800	1.2823	0.9795
Space group	*P*2_1_2_1_2_1_	*P*2_1_2_1_2_1_	*P*2_1_2_1_2_1_	*P*2_1_2_1_2_1_
*a*, *b*, *c* ()	53.14, 66.55, 144.05	53.13, 66.38, 143.79	52.93, 66.67, 144.00	53.17, 66.57, 144.39
Resolution range ()	30.01.60 (1.631.60)	50.01.55 (1.581.55)	50.01.41 (1.431.41)	30.01.60 (1.631.60)
Total No. of reflections	686326 (33531)	375793 (19100)	475587 (19787)	1655884 (81493)‡
No. of unique reflections	68532 (3387)	71540 (3537)	94978 (4497)	131002 (6572)‡
Multiplicity	10.0 (9.9)	5.3 (5.4)	5.0 (4.4)	12.6 (12.4)‡
Completeness (%)	99.9 (100.0)	95.7 (96.1)	95.7 (91.9)	100.0 (100.0)‡
*I*/(*I*)	47.0 (5.7)	31.5 (4.1)	36.6 (2.1)	39.5 (4.6)‡
Wilson *B* factor (^2^)	27.0	24.3	25.1	24.9
*R*_merge_ § (%)	7.0 (53.1)	8.5 (58.9)	7.7 (75.8)	10.1 (68.7)‡
*R*_r.i.m._ ¶ (%)	7.4 (56.0)	9.5 (65.2)	8.5 (85.5)	10.5 (71.6)‡
*R*_p.i.m._ †† (%)	2.3 (17.7)	4.0 (27.3)	3.7 (38.6)	3.0 (20.2)‡
SAD phasing				
Figure of merit (before/after density modification)				0.39/0.64
Model refinement				
PDB code	4q6m	4q6n	4q6o	
No. of Csd4 monomers in asymmetric unit	1	1	1	
Resolution range ()	20.01.60	20.01.55	20.01.41	
*R*_work_/*R*_free_ ‡‡ (%)	19.4/22.0	20.0/22.7	18.9/21.2	
No. of non-H atoms				
Total	3812	3860	4114	
Protein	3366	3366	3366	
Water O	437	446	708	
Glycerol	6	18	24	
Calcium ion	3	3	3	
Muramyltripeptide		27		
*m*DAP			13	
Average *B* factor (^2^)				
Overall	20.2	19.6	23.2	
Protein	18.9	18.1	19.9	
Water O	30.1	29.8	38.0	
Glycerol	39.5	39.9	48.5	
Calcium ion	16.3	17.5	2.3	
Muramyltripeptide		30.1		
*m*DAP			19.9	
R.m.s. deviations from ideal geometry				
Bond lengths ()	0.009	0.008	0.007	
Bond angles ()	1.35	1.32	1.31	
R.m.s. *Z*-scores§§				
Bond lengths	0.435	0.404	0.339	
Bond angles	0.618	0.596	0.596	
Ramachandran plot¶¶ (%)				
Favoured/outliers	97.4/0.0	97.6/0.0	97.7/0.0	
Poor rotamers¶¶ (%)	0.3	0.8	1.0	

† PF, Photon Factory, Japan; PLS, Pohang Light Source, Republic of Korea.

‡ Friedel pairs were treated as separate observations.

§ *R*_merge_ = 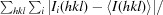 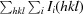, where *I*(*hkl*) is the intensity of reflection *hkl*, 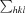 is the sum over all reflections and 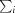 is the sum over *i* measurements of reflection *hkl*.

¶ *R*_r.i.m._ = 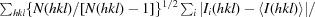 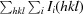.

†† *R*_p.i.m._ = 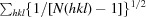 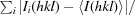 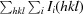.

‡‡ *R*_work_ = 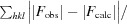 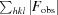, where *R*_free_ is calculated for a randomly chosen 5% of reflections which were not used for structure refinement and *R*_work_ is calculated for the remaining reflections.

§§ Values were obtained using *REFMAC*.

¶¶ Values were obtained using *MolProbity*.

## Footnotes

1 Supporting information has been deposited in the IUCr electronic archive (Reference: MH5144).
