# Supplementary material for: Structural basis for the recognition of muramyltripeptide by Helicobacter pylori Csd4, a d,l-carboxypeptidase controlling the helical cell shape
Source: Acta Crystallogr D Biol Crystallogr. 2014 Oct 16;70(Pt 11):2800–12. doi: 10.1107/S1399004714018732 (PMC4220969; doi:10.1107/S1399004714018732)
Supplement: Supplementary file 1 [file d-70-02800-sup1.pdf]

Acta Crystallographica Section D

Volume 70 (2014)

Supporting information for article:

Structural basis for the recognition of muramyltripeptide by *Helicobacter pylori* Csd4, a D,L-carboxypeptidase controlling the helical cell shape

Hyoun Sook Kim, Jieun Kim, Ha Na Im, Doo Ri An, Mijoon Lee, Dusan Hesek, Shahriar Mobashery, Jin Young Kim, Kun Cho, Hye Jin Yoon, Byung Woo Han, Byung Il Lee and Se Won Suh

Table S1 Statistics on data collection and refinement

A. Data collection		
Data set	Zn1	Zn2
Space group	$P2_12_12_1$	$P2_12_12_1$
Unit cell lengths (Å)	45.5, 66.1, 142.0	53.3, 66.9, 144.8
X-ray wavelength (Å)	1.2823	1.2823
Resolution range[a,b] (Å)	50.0–2.60 (2.64–2.60)	50.0–2.40 (2.44–2.40)
No. total reflections[a,b]	176,100 (8,669)	315,647 (15,050)
No. unique reflections[a,b]	27,639 (1,376)	38,851 (1,905)
Completeness[a,b] (%)	100.0 (100.0)	99.8 (99.8)
$\langle I \rangle / \langle \sigma_I \rangle$ [a,b]	23.2 (3.5)	43.2 (10.1)
Wilson B factor ($Å^2$)	55.9	46.6
R_{merge}[a–c] (%)	12.2 (73.7)	13.0 (60.1)
B. Model refinement		
PDB code	4Q6P	4Q6Q
Resolution range (Å)	30.0–2.62	30.0–2.40
R_{work} / R_{free}[d] (%)	18.4 / 25.1	16.7 / 20.5
No. of non-hydrogen atoms / average B-factor ($Å^2$)		
Total	3,533 / 45.2	3,606 / 33.0
Protein	3,366 / 45.2	3,366 / 32.7
Water oxygen	145 / 42.6	223 / 36.9
mDAP	13 / 58.7	13 / 36.9
Zinc ion	8 / 79.1	2 / 67.9
Calcium ion	1 / 49.5	2 / 40.7
R.m.s. deviations from ideal geometry		
Bond lengths (Å)	0.010	0.009

Bond angles (°)	1.30	1.34
Ramachandran plot (%)[e]		
Favored / Outliers	94.9 / 0.0	96.1 / 0.0
Poor rotamers (%)[e]	3.25	0.54

[a] Values in parentheses refer to the highest resolution shell.

[b] Friedel pairs were treated as separate observations.

[c] $R_{\text{merge}} = \Sigma_h \Sigma_i \mid I(h)_i - \langle I(h) \rangle \mid / \Sigma_h \Sigma_i I(h)_i$, where $I(h)$ is the intensity of reflection h, Σ_h is the sum over all reflections, and Σ_i is the sum over i measurements of reflection h.

[d] $R_{\text{work}} = \Sigma \mid |F_{\text{obs}}| - |F_{\text{calc}}| \mid / \Sigma |F_{\text{obs}}|$, where R_{free} is calculated for a randomly chosen 5% of reflections, which were not used for structure refinement and R_{work} is calculated for the remaining reflections.

[e] Values obtained using *MolProbity*.

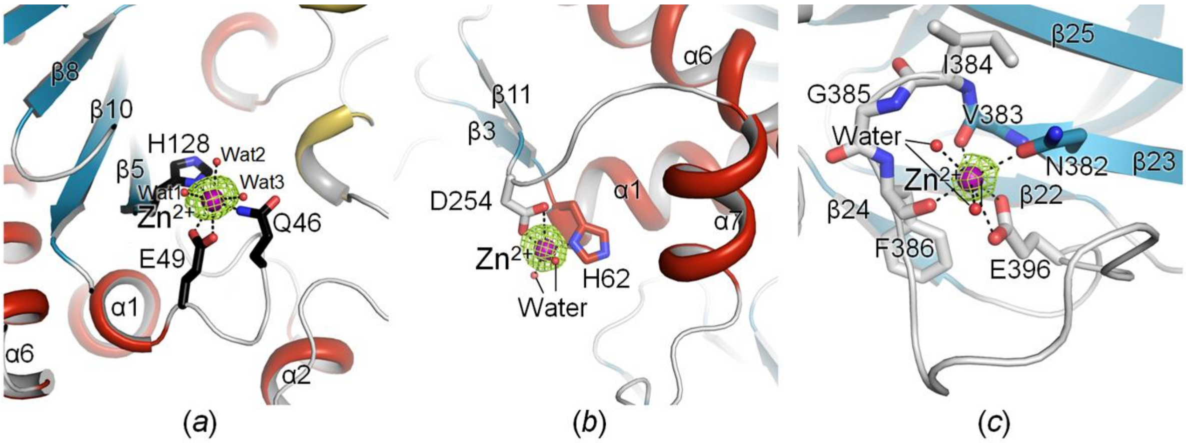

~~**Figure S1**~~ Anomalous difference Fourier maps (contoured at 5σ and colored in green) for Zn^{2+}-replaced metal sites. (*a*) The active site metal ion for D,L-carboxypeptidation by the CPase domain from the Zn1 data set (Table S1). (*b*) The metal ion that stabilizes the β11-α7 loop in the CPase domain from the Zn1 data set (Table S1). (*c*) The metal ion in the calcium-binding site of the C-terminal Ig-like domain from the Zn2 data set (Table S1). The ribbon diagram of metal-binding sites are colored as in Figure 1(*a*).

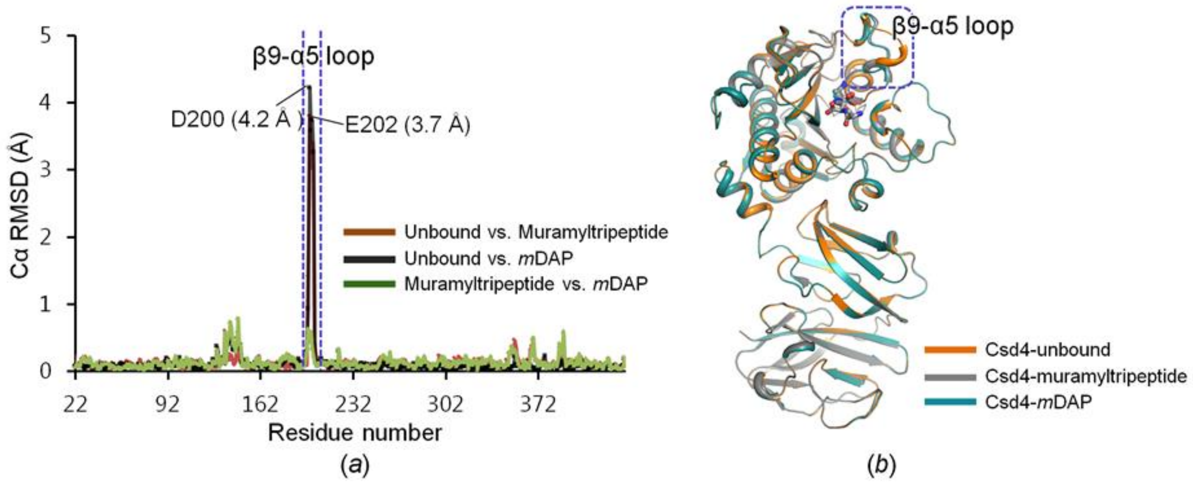

Figure S2 Comparisons of three Csd4 structures. (*a*) A plot of the Cα r.m.s. deviations for pairwise comparisons among Csd4-unbound, Csd4-muramyltripeptide, and Csd4-*m*DAP structures. (*b*) A superposition of three Csd4 models. The Csd4-unbound, Csd4-muramyltripeptide, and Csd4-*m*DAP structures are colored in orange, gray, and cyan, respectively. The β9-α5 loop region is highlighted by the dotted blue box, showing a significant conformational change.

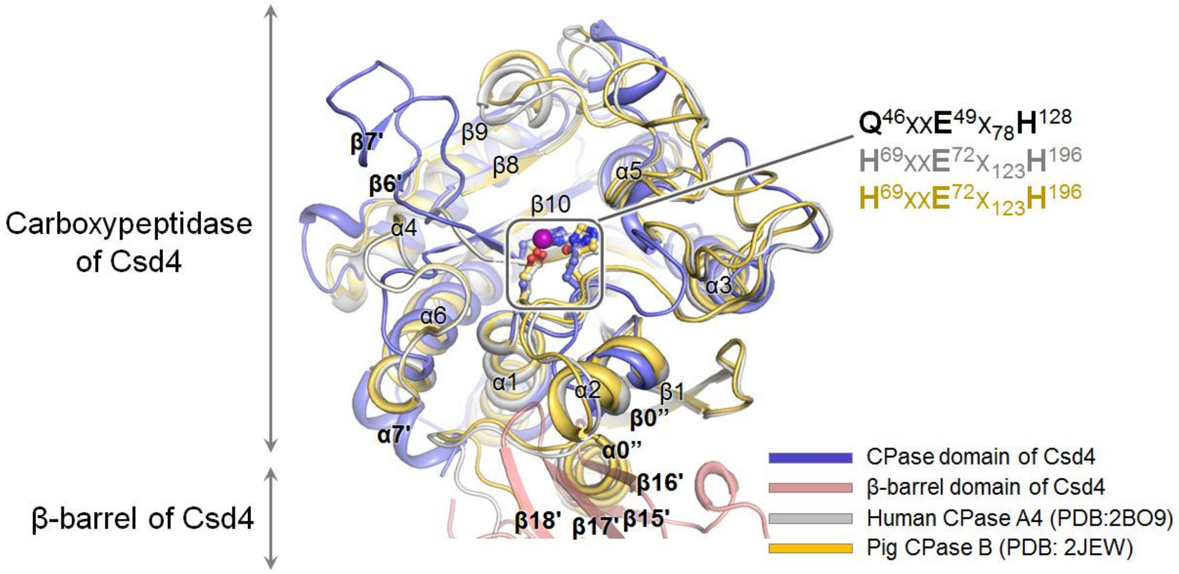

Figure S3 Superposition of carboxypeptidase (CPase) domains in three M14 metallopeptidases. The CPase domains of *H. pylori* Csd4 (bluish-violet), human CPase A4 (gray; PDB code: 2BO9), and pig CPase B (yellow; PDB: 2JEW) are superimposed and shown in ribbon diagrams. The β-barrel domain of *H. pylori* Csd4 is colored in pink. The regions present only in *H. pylori* Csd4 are labeled with a single quote (α7' and β15–β18'), whereas the regions absent only in *H. pylori* Csd4 are labeled with a double quote (α0” and β0”).
